# The Institutionalization of Hatred Politics in the Mediterranean: Studying Corpora of Online News Portals During the European ‘Refugee Crisis’

**DOI:** 10.1007/s11245-023-09890-w

**Published:** 2023-02-08

**Authors:** Dimitris Serafis, Franco Zappettini, Stavros Assimakopoulos

**Affiliations:** 1 Institute of Argumentation, Linguistics and Semiotics, USI–Università della Svizzera italiana, Lugano, Switzerland; 2 Department of Communication and Media, University of Liverpool, Liverpool, UK; 3 Institute of Linguistics and Language Technology, University of Malta, Msida, Malta

**Keywords:** Hatred, Institutionalization, ‘Refugee crisis’, Mediterranean, Topoi/argumentation, Corpus-assisted critical discourse studies

## Abstract

This paper aims to study the argumentative basis on which the prevention of migration is justified and hatred politics is institutionalised in three Mediterranean settings, namely Greece, Malta, and Italy, that were at the centre of the so-called ‘refugee crisis’ in 2015–2017. Following the rubric of corpus-assisted Discourse-Historical Approach (DHA) to Critical Discourse Studies (CDS), we trace (a) the main meaningful patterns, and (b) discursive and argumentation strategies (topoi) in three balanced corpora of mainstream news portals aligned with centre-right and centre-left political views. Among our main findings, the mobilisation of migrant populations is construed as an extremely polarised issue both in national and EU contexts and claims in favour of its prevention are justified on topoi of *danger/threat*, *numbers* and *burdening*/*weighing down*.

## Introduction

Since the increase of the movement of refugee and migrant populations from war and conflict zones in the Middle East to the Mediterranean in summer 2015, the European public sphere has witnessed high polarization (Bevelander and Wodak 2019; Serafis et al. 2021a)﻿. The ‘refugee crisis,’ as this movement was construed by dominant institutions, gave rise to a severe conflict between humanitarian and discriminatory discourses in the European Union (henceforth EU) (Krzyżanowski et al. 2018). In this context, some right-wing and populist public actors in several European contexts discursively mobilised migrant populations for specific political agendas (Krzyżanowski 2018; Cap 2019; Boukala 2021), adding to a general public sentiment of hostility, which was further amplified by a large section of the EU’s mediascape (e.g., Colombo 2018).

Recognising the increase of hatred violence and aggression towards migrants in the EU (ECRI 2017), several EU member-states have introduced laws against hate speech (Alkiviadou 2017) to address the cases when a *public and explicit incitement to violence and/or hatred* is witnessed *against a group–or a member of a group–with protected characteristics* (Council of the European Union 2008). However, these public and explicit cases of incitement to hatred have been seen as constituting marginalized instantiations of hatred in public debates, since, in most of the cases, hate speech “can be concealed in statements which at a first glance may seem to be rational or normal” (Weber 2009, 5). From that point of view, it seems reasonable to assume that “explicitness cannot be the only determining criterion in the identification of hate speech” (Assimakopoulos 2020, 179), which means that we need rigorous tools and methods that can unravel the *raison d’être* of discriminatory discourses and can show how hatred emerges and is justified (Serafis 2022) rather than simply examining any explicit discursive manifestations that fall under a normative remit. This study thus makes sense of media representations of immigration through the lens of “soft hate speech” that is ‘oblique’ incitement to hate, or language which is “lawful but aimed at intolerance and discrimination” (Assimakopoulos et al. 2017, 4; see also Serafis and Assimakopoulos in preparation).

Starting off with the presumption that discriminatory discourses and/or hatred end up framing politics in contemporary societies (Wodak 2015, 2017, 2021), this study illuminates the argumentative basis on which *soft hate speech* may be discursively (re)produced leading to an *institutionalization* (Zappettini in prep) of politics against migrant populations. To this end, we study balanced corpora of articles from mainstream online news portals in Greece, Malta and Italy, which have been identified as the EU member-states that constitute the primary entry points of migrant populations, and have, as a result, experienced highly politicised domestic debates over the ‘refugee crisis’ (European Parliament 2019).

In what follows, we firstly provide a theoretical discussion of the process of institutionalization and the role of mass media in framing politics (Sect. 2). In Sect. 3, we outline our methodological framework which is grounded in the tenets of Critical Discourse Studies (henceforth CDS) and operationalised at two levels. We initially employ tools from Corpus Linguistics (henceforth CL) to scrutinise the main discursive patterns in three large corpora of centre-left and centre-right online news portals. We subsequently zoom in on an ultimate study of the argumentation strategies (i.e., *topoi*), outlined by the Discourse-Historical Approach (henceforth DHA), which can be seen as underlying the main reasoning patterns that justify hatred against migrant populations in the perceived peak of the ‘refugee crisis’ (06/2015–12/2017). Our analysis follows in Sects. 4 and 5. In light of the analysis, we will draw some conclusions in Sect. 6.

## The Institutionalization of (Hatred) Politics

We ground our study within the larger theoretical framework of institutionalization in the “networked public sphere” (Friedland et al. 2006), which is made up of three interrelated social actors: political institutions, mass media and the networked publics. Political institutions refer to those bodies such as the government and parliament which, in democratic systems, are seen as legitimate actors entrusted to provide a legal framework around issues like migratory phenomena. Mass media represent influential institutions of their own as well as communicative channels which, along informative functions, perform important civic functions and can heavily influence public opinion. The networked publics connote an understanding of traditional audiences as enabled by new technology including their ‘prosumers’ function (boyd 2010)–that is not only their ability to consume media discourses but also to (re)produce them. These three actors are entangled in a communicative web which works in mutually constitutive ways in the construction and legitimation of social reality (e.g., Vaara 2014; Lundby 2009; Mazzoleni and Schultz 1999; Zappettini et al. 2021).

Our study specifically focuses on the role of the mass media. By ‘informing’ us about the world, media discourses tend to privilege certain causal readings of issues and certain identities over others, which often leads to framing events and actors around specific interests and set agendas (McCombs 2009; Entman 2010; Edgar 2008; Happer and Philo 2013). This way, not only do the media represent influential actors in the (re)production of discursive logics but they also contribute to the construction of social-cognitive models through which public opinion make sense of political and social phenomena and upon which citizens exercise political agency (McCombs 2009; van Dijk 2008; Scheufele 2000). In other words, media discourses can significantly influence ways of political action in society (Patrona 2018; see also De Rycker and Don 2013).

Of course, the fact that the media have the ability to gatekeep information and to instigate polarised public debates may resonate with different audiences in different and complex ways with no ultimate consensus on whether the network publics perform passive or active roles (Aalberg et al. 2018). It is thus important to see the media as part of a legitimation discursive chain (Zappettini in prep; Zappettini and Bennett 2022) in which discursive practices are “enchained” and diffused by different actors across different genres and social fields (Fairclough 2003). These can gradually sediment into normalised constructions and effectively constitute the argumentative basis upon which a *status quo* may be (de)legitimised. In this sense, discursive chains can become self-reinforcing and path-dependent processes, as specific narratives are given epistemic value over others along the chain. For example, Krzyżanowski (2018) outlines the dynamics of discursive trajectories by tracing discursive shifts leading up to socially normalised scenarios in the context of the Polish immigration debate.

Importantly, as previously mentioned, not only can the discursive framing operated by the mass media over a topic such as immigration contribute to prime audiences’ perceptions of it, but it can also legitimise political decision-making processes triggering a further setting of political agendas and policy deliberations (Kingdon 2014). From an institutional perspective, incremental changes in public sentiment on a topic (which can straightforwardly be seen as driven and reflected by discourses) will thus tend to build up and coalesce in critical junctures (Capoccia 2016, 89) in which “decisions of important actors are causally decisive for the selection of one path of institutional development over other possible paths”. In this sense, while a discourse (and the reasoning that it brings to the fore; see Amossy 2009a, b) can manifest itself synchronically (at one point in time), discursive trajectories can be traced diachronically as the accumulation of various discourses by various actors (see for example Zappettini in prep who, in relation to Brexit, traces distinct discursive phases of *prelegitimation, institutionalization*, and *legacy*). Even though a direction of causality between a newspaper’s political alignment, news coverage and the political attitudes of its readership cannot be established in a linear fashion, we can see public discourses as manifested in the media as capable of prelegitimising and institutionally reinforcing specific (hatred) arguments of the political debate over migration.

Our analysis (as detailed below) therefore treats journalist practices as “an argumentative discourse genre” (Richardson 2007, 64) that combines factual and evaluative content to frame phenomena along certain moral and rational bases and to construct newsworthiness for their audiences, all the while suggesting political actions such the prevention of the migratory phenomenon or justifying a general derogatory and hateful attitude against migrant populations in the examined timeframe and social settings.^1^

## Corpus-Assisted DHA to CDS

As already mentioned before, our study draws on CDS. According to this framework, discourse and social reality are in mutual constitution (Fairclough 1992) and, therefore, public discursive constructions can (re-)produce–or oppose the (re)production of–social inequalities, due to the ideological beliefs these constructions endorse (Flowerdew and Richardson 2018). Studies belonging to CDS “take explicit position, and thus want to understand, expose, and ultimately resist social inequality” (van Dijk 2001, 352) by embracing “standards of careful, rigorous, and systematic analysis” (Fairclough and Wodak 1997, 259), while facilitating a research project that engages with scholarly critique (Reisigl and Wodak 2001) against social injustice (see for example Forchtner 2011 who strengthens CDS’s scholarly critique on the Frankfurt School and Habermas’s thought). We will elaborate on this point in light of our analysis (Sect. 6). To unveil the ways in which ideological beliefs are reproduced in text and talk, CDS scholars examine how *micro-level* textual choices and strategies interrelate with *macro-level* dominant values and views, in a way that ends up supporting (or opposing) them (van Dijk 2008, 85–89). Against this backdrop, our micro-level analysis aims to show how textual choices/strategies in online news portals’ articles provide the meaningful base and the reasoning lines for dominant macro-level racist discriminatory values and views to be justified, institutionalizing anti-migrant and hatred politics in the ‘refugee crisis’-hit Mediterranean.

Our overall dataset comprises online news portals’ articles in three national contexts, namely Greece, Malta and Italy during the peak period of the ‘refugee crisis’ (2015–2017). We selected two mainstream online portals, i.e. portals with high traffic but different ideological orientations, in each national context. More specifically, our data come from the Greek portals *Próto Théma* (henceforth *Théma*; centre-right) and *Efimerída ton Syntaktón* (henceforth *EfSyn*; centre-left), the Maltese *Malta Independent* (henceforth *Independent*; centre-right) and *Malta Today* (henceforth Today; centre-left), and the Italian *Corriere della Sera* (henceforth *Corriere*; centrist/centre-right) and *La Repubblica* (henceforth *Repubblica*; centre-left).

We downloaded all the news articles comprising the terms refugee* and migrant*/immigrant* in all three languages that were published online from 06/2015 to 12/2017, using the European Commission’s platform *NewsBrief*.^2^ In order to polish the initial datasets, we deleted broken links and/or unrelated news articles (e.g., bird migration). We, thus, ended up with a dataset of 507,843 words (1,185 news articles) for Greece, 905,760 words (1,648 news articles) for Malta and 1,239,426 words (1,993 news articles) for Italy.

To ensure representativeness between each national context and news portal, we created three balanced corpora of approximately 100,000 words for each country that is 113,474 words for Greece, 92,437 words for Malta, and 98,715 words for Italy, by randomly incorporating in it one out of every ten or twelve articles for every online news portal, respectively. Thus, we are confident that our balanced corpora constitute a representative sample of the mainstream ideological orientations (i.e., centre-right vs. centre-left) in the Greek, Maltese and Italian mediascape, throughout the topical two-years period of the emergence of the ‘refugee crisis’ in the Mediterranean. Table 1 provides an overview of our corpus.

**Table 1:** Balanced corpora composition

News portal	Country	Timeframe	Words
*Théma*	Greece	06.2015 – 12.2017	55,606
*EfSyn*	Greece	06.2015 – 12.2017	60,018
Total–Greece			113,474
*Independent*	Malta	06.2015 – 12.2017	45,876
*Today*	Malta	06.2015 – 12.2017	46,561
Total–Malta			92,437
*Corriere*	Italy	06.2015 – 12.2017	50,020
*Repubblica*	Italy	06.2015 – 12.2017	48,695
Total–Italy			98,715

In order to analyse our data, we drew on the methodological outlook of corpus-assisted CDS, which enables the researcher to identify the main attitudes that emerge in large corpora within a specific timeframe, all the while avoiding possible biases of data cherry-picking through an automatic software analysis (see Baker 2012; Baker et al. 2008). Following this line of research, we used the *Sketch Engine* software^3^ and we relied on “frequency”, “keywords” and “concordance” analyses (Baker et al. 2013). This methodological continuum enabled us to, firstly, identify both the most frequent words used in our corpus (i.e., frequency analysis), and the most frequent words that are specific to it when cross-examined against other corpora automatically generated by *Sketch Engine* (i.e., keywords analysis). In this way, we were able to pinpoint the main meaningful lines around which online news portals constructions on the ‘refugee crisis’ revolve in the Mediterranean mediascape. Following this mapping of the main meaningful constructions revolving around the phenomenon of migration towards the Mediterranean in the examined timeframe (i.e., frequency and keyword analysis), the concordance analysis paved the way to a more qualitative this time investigation of the portrayals of the targeted groups in (con)text. Specifically, while analysing concordances generated by *Sketch Engine* we were able to initially show how refugee/ migrant populations and their actions are discursively constructed in clause complexes that constitute the concordance lists.

Overall then, starting from a broader investigation of the discursive mobilisation of the event (i.e., migration) in our frequency and keyword analysis, we zoom in on the examination of the main patterns of the discursive mobilisation of the main social actors (i.e., migrants/ refugees) and the actions they undertake or are undergone in this event, through concordances analysis, during which we qualitatively analyse exemplary cases-extracts that represent the main patterns identified (see Sect. 5).

During this step, we specifically drew on a study of “discursive strategies” outlined by the DHA (Reisigl and Wodak 2016). In particular, we studied (a) “nomination strategies”, (b) “predication strategies” and (c) “argumentation strategies” (see Reisigl and Wodak 2016, 33). According to the DHA, *nomination strategies* allow us to examine the “discursive construction of social actors, objects, phenomena, events, processes and actions” usually realised by “metaphors, metonymies [and/or] verbs and nouns [that are] used to denote processes and actions”; *predication strategies* permit us to show “the discursive qualification of social actors, objects, phenomena, events, processes and actions (positively or negatively)” in terms of “(stereotypical) evaluative attributions of negative or positive trails […], explicit predicates or predicative nouns/adjectives/pronouns” (ibid.). This cross-examination gave us a first look at the discursive construction and evaluation of migrant populations in exemplary extracts, which are part of the first twenty concordances appearing in the relevant automatic analysis.

Following the premises of the DHA and relevant recent studies, we argue that specific claim-argument(s) couplings can emerge on the basis of this discursive construction and evaluation (i.e., nomination- and predication strategies) of migrant populations in online news, instantiating certain argumentative *topoi/loci* (see Serafis et al. 2021a, b; see also below for the relevant discussion). These in turn implicitly justify the prevention of migration and the exclusion of minorities from the host societies based on denigrating, discriminatory portrayals of the relevant populations, which, however, do not directly incite to hatred or violence against them, and thus constitute *soft* rather than *hard* hate speech (see Assimakopoulos et al. 2017). To study the argumentative basis on which such claims-arguments may be retrieved, we then move towards a scrutiny of the “argumentativity” (Reisigl and Wodak 2016, 27) of these discursive constructions which we examine in terms of the “argumentation strategies” that outline the “justification and questioning of claims of truth and normative rightness” through the use of *topoi* (see Reisigl and Wodak 2016, 33).

Selectively drawing on Argumentation Theory (e.g., Kienpointner 1997, 2001; Rubinelli 2009), DHA perceives topoi “as the *place* in which an argument is developed” (Boukala 2016, 252, original emphasis) that encapsulate “content-related warrants or ‘conclusion rules’ which connect the argument(s) with the conclusion, the claim[;] justify[ing] the transition from the argument(s) to the conclusion [via] conditional or causal paraphrases such as ‘if x, then y’ or ‘y, because x’” (Reisigl and Wodak 2016, 35; see also Reisigl and Wodak 2001, 75–80). In other words, topoi are the argument(ation) schemes that can be derived from discursive constructions (see nomination and predication strategies, as explained above) and grasp the logical argumentative move made in terms of different conditionals to lead us to a possible claim. This is the logical argumentative basis on which hatred politics against migrant populations can be justified and further institutionalised through their dissemination in the mediascape.

In other words, by analysing the main *argumentation strategies (topoi)* emerging in our corpora, we showcase the ‘logical lines’ on the basis of which claim-argument(s) pairs against migrants/migration can be legitimately developed. It is essential to highlight that we do not claim that the relevant *topoi* constitute hate speech per se; rather, they can legitimately be the *argumentative lieu* on which hatred and exclusion of migrants can be justified all the while being proliferated through media lens (online news portals in our case) across times (2015–2017) and places (Greece, Malta, Italy), institutionalising relevant policies that further establish hatred and exclusionary perspectives in society. In this respect, we claim that the incitement to hatred can be oblique (see also Sect. 1) and therefore the ways this hatred is argumentatively justified should be detected and deciphered in utterances where it is not formulated in an explicit way.

## Main Attitudes in Our Corpora^4^

Starting with an analysis and interpretation the most frequent words appearing in our Greek corpus, we witness a construction of the migration as a highly polarised issue both for the country and its relations with neighbour countries and the EU. Apart from migrant* and refugee*, words such as *Greece, country, island, Lesvos, Europe, EU, Turkey* are very frequently encountered in both corpora coming from the centre-right and the centre-left portals under study (Tables 2 and 3). Then, the high-polarisation is evident through the frequent presence of words that can trigger both humanitarian (see *hospitality, child, asylum*–Table 2/*Théma*; *right, asylum*–Table 3/*EfSyn*) and discriminatory (see *centre, crisis, border*–Table 2/*Théma*; *centre, border, detention*–Table 3*/EfSyn*) attitudes in relation to migration. The same conflict between humanitarian vis-à-vis discriminatory attitudes becomes more evident when one looks at the keyword tables (Tables 4 and 5). There, keywords (i.e., both nouns and adjectives) related to migration appear together with lemmas such as *hospitality* versus *camp* and *expulsion*. All in all, this preliminary analysis confirms that the ‘refugee crisis’ provided fertile ground for the co-existence of discriminatory and humanitarian stances in media discourse surrounding migration that was discussed in Sect. 1.

**Table 2:** Frequency–*Théma*

Rank	Word	English translation	Freq. count
1	Πρόσφυγας	Refugee	535
2	Μετανάστης	Migrant	363
3	Χώρα	Country	203
4	Νησί	Island	181
5	Ελλάς	Hellas/Greece	176
6	Κέντρο	Centre	172
7	Ευρωπαϊκός	European	162
8	Ελληνικός	Greek	153
9	Φιλοξενία	Hospitality	111
10	Κατάσταση	Situation	109
11	Κυβέρνηση	Government	106
12	Ευρώπη	Europe	103
13	Παιδί	Child	102
14	Υπουργός	Minister	99
15	Άνθρωπος	Person	97
16	Άσυλο	Asylum	93
17	Καταυλισμός	Camp	92
18	Κρίση	Crisis	91
19	Σύνορο	Border	90
20	Περιοχή	Area	84
21	Χώρος	Site	84
22	Αρχή	Authority	83
23	Τουρκία	Turkey	81
24	Λέσβος	Lesvos	74
25	EE	EU	74

**Table 3:** Frequency–*EfSyn*

Rank	Word	English translation	Freq. count
1	Πρόσφυγας	Refugee	586
2	Μετανάστης	Migrant	279
3	Ελλάς	Hellas/ Greece	167
4	Δικαίωμα	Right	161
5	Νησί	Island	157
6	Κέντρο	Centre	154
7	Άσυλο	Asylum	142
8	Χώρα	Country	134
9	Ευρωπαϊκός	European	123
10	Σύνορο	Border	121
11	Άνθρωπος	Person	118
12	Λέσβος	Lesvos	117
13	Τουρκία	Turkey	116
14	Διεθνής	International	103
15	Κυβέρνηση	Government	102
16	Ελληνικός	Greek	101
17	Πολιτική	Politics	99
18	Ευρώπη	Europe	98
19	Οργάνωση	Organization	93
20	Φιλοξενία	Hospitality	88
21	Πολιτικός	Politician	88
22	Υπουργός	Minister	86
23	Διαδικασία	Procedure	84
24	Συμφωνία	Agreement	83
25	Κράτηση	Detention	79

**Table 4:** Keywords–*Théma*

Rank	Keyword	English translation	Freq. (F)	Freq. (R)	Relative freq. (F)	Relative freq. (R)	Score
1	Mουζάλας	Mouzalas	49	65	765.55322	0.03317	741.95
2	Προσφυγικός	refugee (adj.)	123	9354	1921.69482	4.77274	333.07
3	Πρόσφυγας	refugee (noun)	535	57,386	8358.5918	29.28035	276.07
4	Καταυλισμός	Camp	92	10,022	1437.36523	5.11358	235.27
5	Φιλοξενία	Hospitality	111	46,335	1734.2124	23.64174	70.418
6	Άσυλο	Asylum	93	38,604	1452.98877	19.69712	70.251
7	Μετανάστης	migrant (noun)	363	159,819	5671.34326	81.54527	68.718
8	Μυτιλήνη	Mytilene	56	23,463	874.91797	11.97165	67.526
9	Λέσβος	Lesvos	74	32,784	1156.1416	16.72755	65.274
10	Μεταναστευτικός	migrant (adj.)	54	23,729	843.6709	12.10737	64.442

**Table 5:** Keywords–*EfSyn*

Rank	Keyword	English translation	Freq. (F)	Freq. (R)	Relative freq. (F)	Relative freq. (R)	Score
1	Προσφυγικός	refugee (adj.)	133	9354	1925.1925	4.77274	333.67
2	Πρόσφυγας	refugee (noun)	586	57,386	8482.42676	29.28035	280.16
3	Απέλαση	Expulsion	60	7603	868.50787	3.87932	178.2
4	Καταυλισμός	Camp	58	10,022	839.55762	5.11358	137.49
5	Άσυλο	Asylum	142	38,604	2055.46875	19.69712	99.36
6	Λέσβος	Lesvos	117	32,784	1693.59045	16.72755	95.591
7	Μυτιλήνη	Mytilene	54	23,463	781.6571	11.97165	60.336
8	Φιλοξενία	Hospitality	88	46,335	1273.81165	23.64174	51.734
9	Μετανάστης	migrant (noun)	279	159,819	4038.56177	81.54527	48.938
10	Σύνορο	Border	121	97,339	1751.49097	49.66578	34.589

A similar picture emerges when one examines the most frequent words and keywords in the Maltese corpus. Irrespective of their ideological orientation, both news portals under study frequently use words such as *EU, country, border, European, asylum* (see Tables 6 and 7), as well as *rescue* (Table 6/*Independent*) or *police* (Table 7/*Malta Today*), which can form the conceptual basis for both discriminatory and humanitarian viewpoints. Moreover, in these corpora, too, the migratory phenomenon is tightly related to neighbour countries (i.e., *Italy* and *Libya*) as evident by the relevant words that appear in both the frequency (Tables 6 and 7) and the keyword analysis (Tables 8 and 9).^5^

**Table 6:** Frequency–*Independent*

Rank	Word	Freq. count
1	Migrant	406
2	Refugee	234
3	EU	232
4	Country	229
5	People	194
6	Malta	160
7	European	144
8	Europe	129
9	Border	128
10	State	115
11	Other	109
12	Minister	108
13	Turkey	105
14	Boat	98
15	Take	97
16	All	92
17	Police	87
18	Need	86
19	Report	83
20	Asylum	82
21	Libya	81
22	Migration	81
23	Italy	79
24	Them	78
25	Rescue	77

**Table 7:** Frequency–*Today*

Rank	Word	Freq. count
1	People	266
2	Migrant	231
3	Malta	220
4	Refugee	216
5	EU	181
6	Country	170
7	Europe	130
8	European	126
9	Asylum	122
10	Child	109
11	Other	105
12	State	97
13	Migration	95
14	Government	94
15	Italy	92
16	Member	86
17	Libya	82
18	Seeker	82
19	Need	80
20	Minister	80
21	Some	79
22	Those	76
23	Border	75
24	Police	73
25	Report	73

**Table 8:** Keywords–*Independent*

Rank	Keyword	Freq. (F)	Freq. (R)	Relative freq. (F)	Relative freq. (R)	Score
1	Malta	160	200,071	3060.5022	4.44909	561.84
2	Migrant	406	636,473	7766.02441	14.15359	512.55
3	Libya	81	325,284	1549.37927	7.23352	188.3
4	Asylum	82	412,241	1568.50745	9.16723	154.37
5	Refugee	234	1,270,097	4475.98486	28.24384	153.09
6	EU	232	2,016,458	4437.72852	44.84107	96.829
7	Migration	81	956,673	1549.37927	21.27406	69.605
8	Turkey	105	1,393,300	2008.45459	30.98357	62.828
9	Rescue	77	1,611,921	1472.8667	35.84516	40.002
10	Italy	79	1,708,480	1511.12305	37.9924	38.78

**Table 9:** Keywords–*Today*

Rank	Keyword	Freq. (F)	Freq. (R)	Relative freq. (F)	Relative freq. (R)	Score
1	Malta	220	200,071	4146.24951	4.44909	761.09
2	Migrant	231	636,473	4353.56201	14.15359	287.36
3	Asylum	122	412,241	2299.28394	9.16723	226.25
4	Libya	82	325,284	1545.42029	7.23352	187.82
5	Seeker	82	353,312	1545.42029	7.85679	174.6
6	Refugee	216	1,270,097	4070.86328	28.24384	139.24
7	Migration	95	956,673	1790.4259	21.27406	80.427
8	Italy	92	1,708,480	1733.88611	37.9924	44.493
9	Europe	130	4,525,568	2450.05664	100.63751	24.116
10	European	126	4,881,254	2374.67017	108.5471	21.686

The frequency and keywords analysis of Italian news portals’ corpora provide us with more insight. While lemmas that are similar to the previous corpora (i.e., *country, Europe, European*, both in Tables 10 and 11) also frequently appear in the Italian one, in this case, there is an additional presence of verbal types that connote the migratory mobilization to and from the host country (see *migrate, arrive*–Tables 10 and 11; *come stay, go*–Table 10/*Corriere*) to other EU member-states, corroborating the view that European South was a transit-place for migrant populations seeking to move to Northern EU member-states. Surprisingly, if one takes into account its centre-left ideological orientation, *Repubblica* frequently uses the discriminatory term *illegal immigrant*, favouring in this sense an anti-migrant portrayal that has been witnessed in recent studies on the 2015–2017 ‘refugee crisis’ in the Italian context (e.g., Serafis et al. 2021a). Similarly, keywords such as *hospitality* (Tables 11 and 12) vis-à-vis *police* or *coast guard* (Table 11/*Corriere*) as well as *crime, illegal immigrant* (Table 12/*Repubblica*), appear to suggest that the Italian corpus leans more towards discriminatory discourse^6^ (see Table 13).

**Table 10:** Frequency–*Corriere*

Rank	Word	English translation	Freq. count
1	Migrare	to Migrate	216
2	Anno	Year	134
3	Italia	Italy	121
4	Altro	Other	119
5	Persona	Person	108
6	Arrivare	to Arrive	107
7	Stare	to Stay	87
8	Italiano	Italian	85
9	Profugo	Refugee	84
10	Andare	Go	83
11	Venire	Come	79
12	Accoglienza	Hospitality	72
13	Centro	Centre	71
14	Libico	Libyan	70
15	Paese	Country	67
16	Ong	NGO	66
17	Libia	Libya	62
18	Nave	Boat	59
19	Governo	Government	56
20	Caso	Case	56
21	Europeo	European	56
22	Chiedere	Ask	56
23	Parlare	Talk	56
24	Centrale	Central	56
25	Europa	Europe	55

**Table 11:** Frequency–*Repubblica*

Rank	Word	English translation	Freq. count
1	Migrare	Migrate	206
2	Anno	Year	144
3	Italia	Italy	131
4	Italiano	Italian	109
5	immigrazione	Immigration	105
6	Stare	Stay	90
7	Clandestino	illegal immigrant	88
8	Paese	Country	83
9	Venire	Come	82
10	Arrivare	Arrive	77
11	Cittadino	Citizen	76
12	Sindaco	Mayor	76
13	Persona	Person	74
14	Presidente	President	70
15	Accoglienza	Hospitality	70
16	Europeo	European	66
17	Governo	Government	66
18	Europa	Europe	63
19	Immigrato	Immigrant	61
20	Giorno	Day	58
21	Nuovo	New	55
22	Parlare	Talk	53
23	Straniero	Foreigner	53
24	Milano	Milan	52
25	Chiedere	Ask	50

**Table 12:** Keywords–*Corriere*

Rank	Keyword	English translation	Freq. (F)	Freq. (R)	Relative freq. (F)	Relative freq. (R)	Score
1	Libico	Libyan	70	27,743	1190.23328	4.73067	207.87
2	Ong	NGO	66	32,081	1122.21997	5.47038	173.59
3	Migrare	Migrate	216	128,584	3672.71997	21.92584	160.24
4	Profugo	Refugee	84	65,332	1428.27991	11.14026	117.73
5	Libia	Libya	62	62,186	1054.20667	10.60381	90.936
6	Asilo	Asylum	48	131,061	816.15997	22.34821	34.999
7	Accoglienza	Hospitality	72	235,515	1224.23999	40.15947	29.768
8	Nave	Boat	59	281,354	1003.19666	47.97582	20.504
9	UE	EU	40	234,234	680.1333	39.94103	16.637
10	Guardia	Police/ Coast Guard	42	253,731	714.13995	43.26561	16.156

**Table 13:** Keywords–*Repubblica*

Rank	Keyword	English translaton	Freq. (F)	Freq. (R)	Relative freq (F)	Relative freq. (R)	Score
1	Migrare	Migrate	206	128,584	3598.00195	21.92584	156.99
2	Salvini	Salvini	45	27,690	785.97131	4.72163	137.54
3	Clandestino	Illegal immigrant	88	69,710	1537.0105	11.88678	119.35
4	Immigrazione	Migration	105	107,428	1833.93298	18.31837	94.984
5	Libia	Libya	44	62,186	768.50525	10.60381	66.315
6	Profugo	Refugee	44	65,332	768.50525	11.14026	63.385
7	Immigrato	Immigrant	61	138,735	1065.42773	23.65676	43.251
8	Accoglienza	Hospitality	70	235,515	1222.62195	40.15947	29.729
9	Lega	League	42	248,547	733.57318	42.38165	16.933
10	Reato	Crime	40	278,728	698.64111	47.52805	14.417

**Fig. 1:**
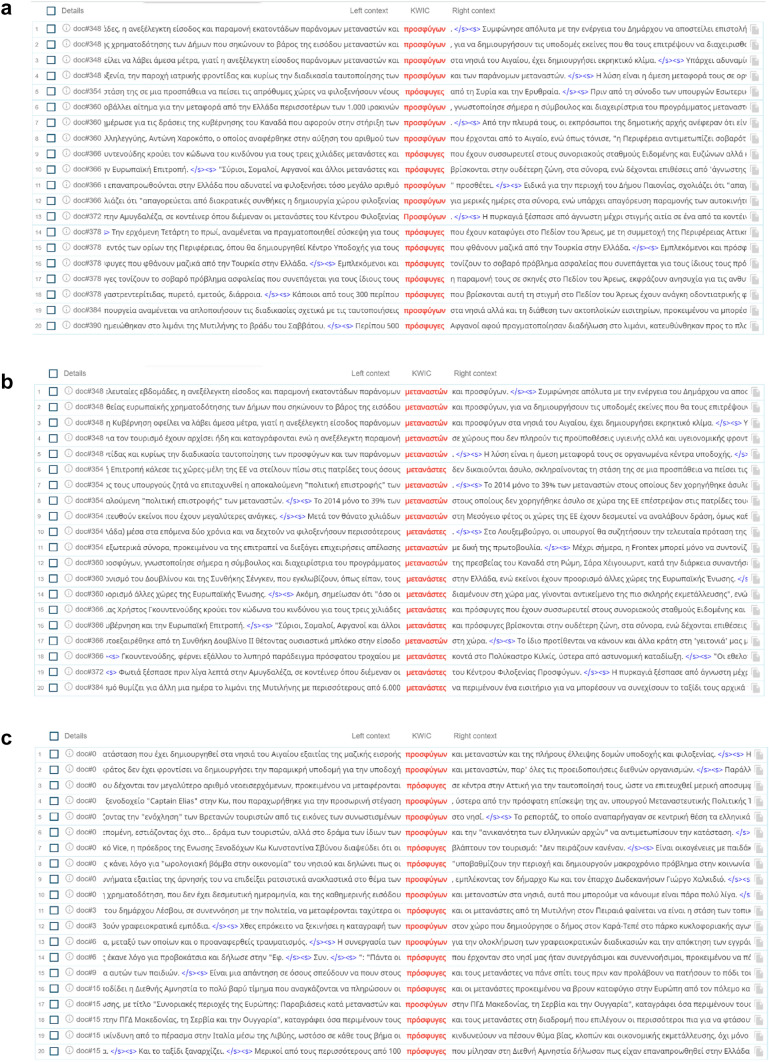

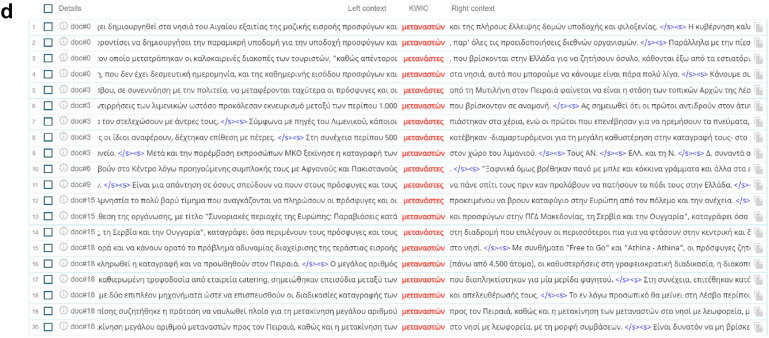
**a** Concordances refugee*–*Théma*. **b** Concordance migrant*–*Théma*. **c** Concordances refugee*–*EfSyn*. **d** Concordances migrant*–*EfSyn*

**Fig. 2:**
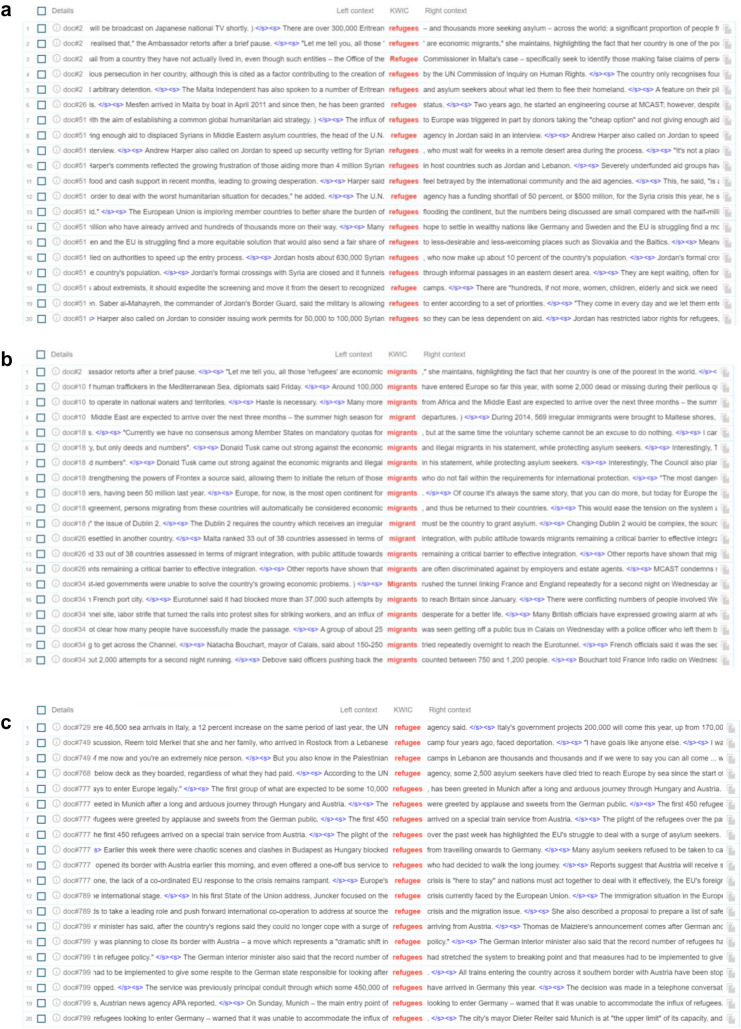

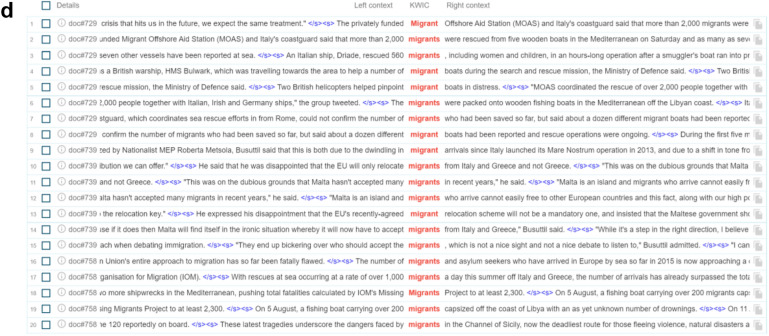
**a** Concordances refugee*–*Independent*. **b** Concordances migrant*–*Independent.* **c** Concordances refugee*–*Today.* **d** Concordances migrant*–*Today*

**Fig. 3:**
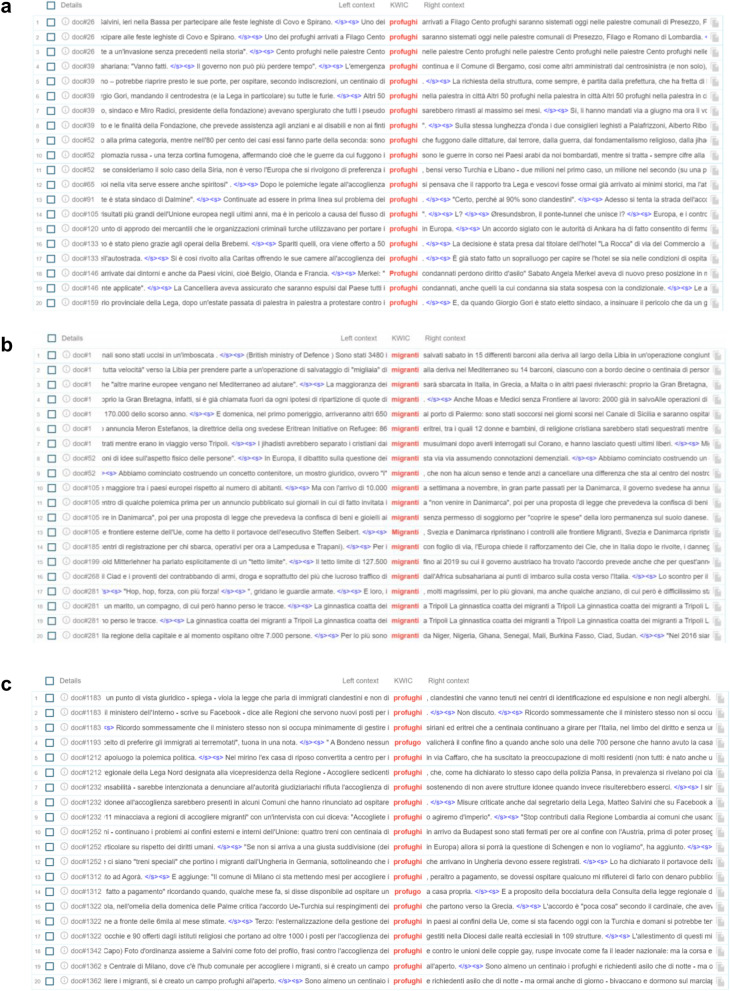

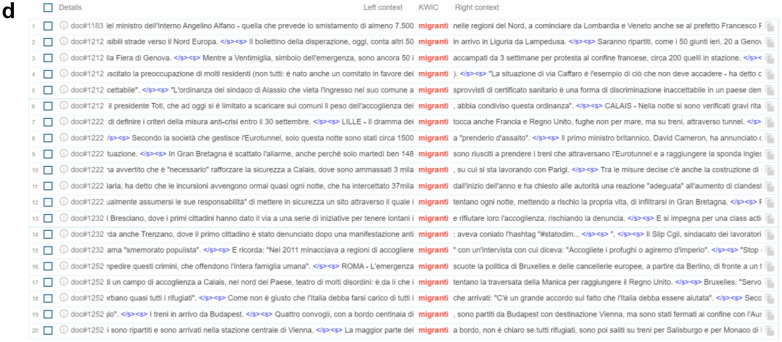
**a** Concordances refugee*–*Corriere*. **b** Concordances migrant*–*Corriere.* **c** Concordances refugee*–*Repubblica*. **d** Concordances migrant*–*Repubblica*

Having gained a preliminary glimpse of the main attitudes in our corpus, we then moved to examine how refugee and migrant populations are construed in (con)text. To that end, we analysed the first 20 out of 100 concordances of the relevant types in our corpora. Figures 1–3 portray these concordances.

Starting with the Greek corpus (Figs. 1a–d), a first, significant, pattern that we identified is that, in many cases, regardless of the ideological orientation of the news portal, migrant and refugee populations are construed as enjoying equal status; this transpires from the fact that the relevant nominal types are linked in parataxis–through the marker *and*–in traced concordances. In this respect, it seems that populations who should be protected by international law (i.e. refugees) are devaluated when assigned equal status to populations who migrate because of economic and/or other reasons. Moreover, the fact that the relevant populations are realized always in plural, gives an impression of a dense group with no individually evident characteristics, shifting the focus from their place of origin and the reasons for their mobilization to their massive presence on Greek soil; a representation that can facilitate the construction of a state of emergency and the necessity for countermeasures (see also Serafis et al. 2020, 551, based on van Leeuwen 2008, 47).

As realised in the concordances coming from *Théma*, we come across a construction of an *uncontrollable* phenomenon, whereby *entrance and stay of illegal immigrants and refugees* is provided along with the necessity for itssss prevention by the EU authorities (see e.g., *the uncontrollable entrance and stay of illegal migrants and refugees* [doc#348]; *the European Commission called EU member-states to send back to their countries those migrants that do not grant the right to asylum* [doc#354]). This need to confine migration is further backed up on constructions that picture a massive number of refugees coming to or staying on Greek soil (e.g., *more than 1,000 Iraqi refugees*, *the increase of refugees coming from the Aegean* [doc#360]; *ring the danger alarm for the three thousands of migrants and refugees who are accumulated in the border stations of Eidomeni and* […] [doc#366]). Overall, the examined concordances provide us with preliminary evidence of the ways migrant and refugee populations are treated by the centre-right news portal in terms of a dense, dangerous and massive social body present in Greece.

Although *EfSyn* is in line with its centre-left orientation, and thus takes a more sympathetic position regarding migration, similar problematic constructions appear here too (see *massive inflows of refugees and migrants*, *images of serried refugees in the island* [doc#0], *huge inflow of migrants in the island* [doc#18]). According to these constructions, migrants are portrayed in terms of dehumanising metaphors (i.e., *inflow*),^7^ while their massive presence in Greece is emphasised (e.g., *massive, huge*. Moreover, in some cases, they are also portrayed as prone to violence (*some migrants were involved into fight* [doc#3], *there were incidents between migrants upon a portion of food* [doc#18]).

Turning to the Maltese news portals (Figs. 2a–d), we notice a distinction between those populations who could be granted the status of international protection (i.e., refugees) and migrant ones. However, the discursive patterns regarding both populations are again similar to the ones retrieved in the Greek corpus, aggravating the sense of an emergency–a ‘crisis’ that has to be prevented–on the basis of existing discriminatory stances that can be identified in the Maltese context (e.g., Assimakopoulos and Vella Muskat 2017).

Specifically, while reporting on refugee populations, the centre-right *Independent* is capitalizing on concordances such as: *There are over 300,000 Eritrean refugees–and thousands more seeking asylum–across the world* [doc#2], *the influx of refugees to Europe was triggered in part by donors* […]*, the burden of refugees flooding the continent* [doc#51], which promote a dehumanised portrayal of the relevant populations (in line with such metaphors as: *influx of refugees*), foregrounding their massive presence (see the relevant numbers) and explicitly construing them as a *burden* in the host soil. Similar patterns appear when looking at the concordances of the word migrant* (see *Around 100,000 of migrants have entered Europe so far this year* [doc#10], *Donald Tusk came out strong against the economic migrants and illegal migrants in his statement* [doc#18], *influx of migrants desperate for a better life* [doc#34]). Apart from the numbers employed to connote a massive presence of migrant populations, nominal types such as *illegal migrants* can also be seen as provoking a discriminatory stance against the respective populations.

The same construction is favoured by the centre-left *Malta Today*, when it comes to reporting on migrant populations, too. Concordances such as *with rescues at sea occurring at a rate of over 1,000 migrants a day* (doc#758), accelerate the sense of emergency because of the burden, i.e., the large numbers of migrant populations that Mediterranean member-states must rescue. This seems to be decreased only in cases when countermeasures are implemented (e.g., […] *due to the dwindling in migrant arrivals since Italy launched its Mare Nostrum operation in 2013* [doc#739]). When it comes to representing refugee populations in this context, once more, reporting on their massive presence creates a sense of emergency (e.g., *The record number of refugees has stretched the system to breaking point* [doc#799]), while even more problematic and unique among the concordances under study, is a construal of their mobilisation in terms of a *crisis* that EU officials pinpoint (see *Junker focused on the refugee crisis currently faced by the European Union* [doc#789]) and the Union has to deal with (see *Europe’s refugee crisis is “here to stay”* [doc#777]).

Finally, in accordance with the Maltese corpus, the concordances analysed from Italian news portals (Figs. 3a–d) make a clear distinction between migrant and refugee populations without creating paratactical linkages, and thus meaningful associations between them, as we witnessed in Greek newspeak.

The centre-right *Corriere* problematises refugees mobilisation by employing terms such as *refugee emergency* or *refugee problem* (see *The refugee emergency continues, […] that all the so-called refugees would remain six months* [doc#39], *on the refugee problem* [doc#91]). On top of that, dehumanising metaphors are used also in this case (see *because of the flux of refugees* [doc#105]), “fram[ing] migration as a threat and thus assign[ing] a special sense of urgency to the situation, indirectly calling for mobilization on the part of the in-group to defend itself” (Serafis et al. 2021b, 570). While portraying migrant populations, the picture does not change much. For example, constructions like *rescue operation of “thousands” of migrants* (doc#1), *the arrival of 10,000 of migrants every week in November* (doc#105), give rise to an interpretation whereby there is a massive mobilisation of migrants to the host territory, while, in other cases the emergent issue of migration is additionally pinpointed (e.g., *In Europe the debate on the migration issue is taking on unbelievable connotations* [doc#52]). All in all, migration is construed as an emergency and a problem, a burden for the host country/ies; a construction that implies a need to prevent it.

On the contrary, the centre-left *Repubblica* seems to mostly empathise with refugee populations, as evident in constructions like *was available to host refugees at home* (doc#1312), […] *criticizes the EU-Turkey agreement for pushing back refugees who depart from Greece,* […] *more than 1,000 places for hosting refugees* (doc#1322). Along with these, however, concordances that aggregate refugees and present them undertaking negatively evaluative actions (see *there are at least a hundred of refugees and asylum seekers who during the night* […] *are drunk* [doc#1362]) are present, in line with previous constructions in different news portals and national settings. This portrayal is further supported by the news portal’s reports on migrants. Despite its centre-left orientation, *Repubblica* also appears to follow discriminatory lines similar to the ones witnessed in previous analyses when migrants were portrayed as a numerous mass (in this instance, consider, *at least 7,500 migrants in regions of the North* [doc#1183], *where 3 thousands of migrants are grouped* [doc#1222], *hundreds of migrants* [doc#1252]) that burdens the host societies (*The burden of hosting migrants* [doc#1212]) and accelerates a sense of emergency (*the migrants’ emergency* [doc#1252]).

In sum, when one looks at concordances that include the main target populations, one can easily witness constructions of threat and burden assigned to them through the use of dehumanising metaphorical vehicles (e.g., *influx, flows*) and/or negative evaluative language (e.g., *emergency, burden, problem, crisis, illegal*). Along with a use of numerals (e.g., *hundreds, thousands* etc.) that pinpoint their massive presence in the host soils, these can be seen to aggravate the sense of emergency that the so-called crisis gives rise to.

## Threat—Burden—Numbers: the Argumentative Basis of Hatred

Following our concordance analysis, in what follows, we will qualitatively examine the tendency of portraying migrant populations as massive, a threat, and a burden. Identifying DHA strategies that emerge in example-case extracts we will show how hatred against migrant and refugees can be solidified and justified through the defense of the prevention of migration.

1. […] για τις εκρηκτικές διαστάσεις και τα προβλήματα που έχει δημιουργήσει στο νησί τις τελευταίες εβδομάδες, η ανεξέλεγκτη είσοδος και παραμονή εκατοντάδων παράνομων μεταναστών και προσφύγων. […] κι ενόψει της έναρξης της τουριστικής περιόδου, η Κυβέρνηση οφείλει να λάβει άμεσα μέτρα, γιατί η ανεξέλεγκτη είσοδος παράνομων μεταναστών και προσφύγων στα νησιά του Αιγαίου, έχει δημιουργήσει εκρηκτικό κλίμα.

[…] for the explosive dimensions and problems that the uncontrolled entry and stay of hundreds of illegal immigrants and refugees has created on the island in recent weeks. [...] and in view of the beginning of the tourist season, the Government must take immediate measures, because the uncontrolled entry of illegal immigrants and refugees to the Aegean islands has created an explosive atmosphere. (doc#348; 6/2/2015, Théma).^8^

(2) Σκηνές, σλίπιν μπαγκ και είδη καθαριότητας, συνολικής αξίας 300 χιλιάδων ευρώ, ετοιμάζεται να στείλει στην Ελλάδα ο διεθνής Ερυθρός Σταυρός, προκειμένου να βελτιωθεί κατ' ελάχιστον η κατάσταση που έχει δημιουργηθεί στα νησιά του Αιγαίου εξαιτίας της μαζικής εισροής προσφύγων και μεταναστών και της πλήρους έλλειψης δομών υποδοχής και φιλοξενίας.

The international Red Cross is preparing to send tents, slip-in bags, and cleaning supplies to Greece, with a total value of 300 thousand euros, in order to improve at least the situation that has been created in the Aegean islands due to the massive influx of refugees and immigrants and the complete lack of reception and hospitality structures. (doc#0; 6/2/2015, EfSyn).^9^

(3) Η νέα κινητοποίηση προσφύγων που φιλοξενούνται στο άτυπο κέντρο υποδοχής του Δήμου Λέσβου στην περιοχή Καρά Τεπέ και ο ωριαίος αποκλεισμός κεντρικού δρόμου της Μυτιλήνης ήρθαν να υπενθυμίσουν για άλλη μια φορά και να κάνουν ορατό το πρόβλημα αδυναμίας διαχείρισης της τεράστιας εισροής μεταναστών στο νησί. […] Ο μεγάλος αριθμός μεταναστών (πάνω από 4.500 άτομα), οι καθυστερήσεις στη γραφειοκρατική διαδικασία, η διακοπή υδροδότησης στην περιοχή, τα προβλήματα σίτισης και ο μικρός αριθμός προωθήσεων προς τον Πειραιά (300 άτομα την ημέρα, σύμφωνα με πληροφορίες) έχουν δημιουργήσει ένα εκρηκτικό κλίμα.

The new mobilization of refugees, who are hosted in the informal reception centre of the Municipality of Lesvos in the Kara Tepe area, and the hourly blockade of the central road of Mytilini came to remind once again and make visible the problem of the inability to manage the huge influx of immigrants to the island. […] The large number of migrants (over 4,500 people), the delays in the bureaucratic process, the interruption of water supply in the area, the feeding problems and the small number of advances to Piraeus (300 people per day, according to information) have created an explosive atmosphere. (doc#18; 7/10/2015, EfSyn).^10^

In extract 1, the targeted populations are paratactically linked (see the marker *and*) and discursively constructed (nomination strategy) through nominal types that connote their massive mobilization (*uncontrollable entry and stay*) and openly discriminatory adjectives (*illegal*). They are moreover qualified, in the relevant predicates, as responsible for *explosive dimensions and problems* or the *explosive atmosphere* that their presence has created. These portrayals can give rise to a reasoning that supports different claims in favour of the prevention of the migratory mobilization, along two interweaved DHA topoi. On the one hand, a *topos of threat or danger*, which is realized in terms of the conditional: “if there are specific dangers and threats, one should do something against them” (see Reisigl and Wodak 2001, 77). On the other, this topos can be further corroborated by a *topos of numbers*, which “may be subsumed under the conclusion rule: if the numbers prove a specific topos, a specific action should be performed” (see Reisigl and Wodak 2001, 79).

Similar to that, in extracts 2 and 3, nominations such as *the massive influx of refugees* (extract 2) and *The large number of migrants* (over 4,500 people) (extract 3), and predicates such as *to improve at least the situation that has been created in the Aegean islands due to the massive influx of refugees and immigrants* (extract 2) and *The large number of migrants (over 4,500 people), […] have created an explosive atmosphere* (extract 3) can be seen to facilitate xenophobic perspectives and claims of hatred against migrant populations; again, along the lines of the two aforementioned topoi of threat/danger and numbers.

(4) Around 100,000 migrants have entered Europe so far this year, with some 2,000 dead or missing during their perilous quest to reach the continent. Dozens of boats are launched from lawless Libya each week, with Italy and Greece bearing the brunt of the surge. […] Many more migrants from Africa and the Middle East are expected to arrive over the next three months – the summer high season for migrant departures. (doc#10; 6/20/2015, *Independent*).^11^
(5) The number of migrants and asylum seekers who have arrived in Europe by sea so far in 2015 is now approaching a quarter of a million, according to an analysis by the International Organisation for Migration (IOM). With rescues at sea occurring at a rate of over 1,000 migrants a day this summer off Italy and Greece, the number of arrivals has already surpassed the total arrivals in 2014, the IOM said in a press release. (doc#758; 8/14/2015, *Today*).^12^

In almost the same vein, in the extracts from the Maltese news portals under study we find predicates like *Dozens of boats are launched from lawless Libya each week, with Italy and Greece bearing the brunt of the surge* (extract 4) or *The number of migrants and asylum seekers who have arrived in Europe by sea so far in 2015 is now approaching a quarter of a million* (extract 5) including nominal types that construct a massive mobilization (*100,000 migrants*, *Dozens of boats* [extract 4], *a quarter of a million* [extract 5]), all the while backing up standpoints in favour of a prevention of migration on a *topos of numbers*, as explained above.

(6) L'emergenza profughi continua e il Comune di Bergamo, così come altri amministrati dal centrosinistra (e non solo), sta lavorando per trovare soluzioni riassumibili nel concetto di "accoglienza diffusa": gruppi poco numerosi spalmati su più alloggi temporanei. Nel frattempo però i continui arrivi obbligano a cercare anche spazi di grande dimensione […] The refugee emergency continues and the Municipality of Bergamo, as well as other ones administered by the centre-left (and not only), is working to find solutions that can be summarized in the concept of "widespread reception": small groups spread over several temporary accommodations. In the meantime, however, the continuous arrivals also force us to look for large spaces […] (doc#39, 7/29/2015, *Corriere*).^13^
(7) Per il momento le strutture di accoglienza reggono le richieste, anche sotto il profilo sanitario, ma dalla frontiera si allontanano molti che cercano altre possibili strade verso il Nord Europa. Il bollettino della disperazione, oggi, conta altri 50 migranti in arrivo in Liguria da Lampedusa. Saranno ripartiti, come i 50 giunti ieri, 20 a Genova, 10 a Savona, 10 alla Spezia e 10 ad Imperia, e ospitati nelle strutture di accoglienza sul territorio.

For the moment, the reception structures are holding up against demands, even from the health point of view, but many are leaving the border looking for other possible routes to Northern Europe. The bulletin of despair today counts another 50 migrants arriving in Liguria from Lampedusa. They will be distributed again, like the 50 arrived yesterday, 20 in Genoa, 10 in Savona, 10 in La Spezia and 10 in Imperia, and hosted in the reception facilities in the area. (doc#1212; 7/3/2015, Repubblica).^14^

(8) Questi disperati per poter varcare il confine avrebbero pagato circa 150 euro ciascuno. I conducenti dei mezzi vistisi scoperti perchè inseguiti dalle forze dell'ordine lasciavano i mezzi in autostrada, con a bordo i clandestini, in pendenza e contromano. Ormai in fondo a via Sammartini, accanto alla stazione Centrale di Milano, dove c'è l'hub comunale per accogliere i migranti, si è creato un campo profughi all'aperto. Sono almeno un centinaio i profughi e richiedenti asilo che di notte—ma ormai anche di giorno—bivaccano e dormono sul marciapiedi e sul prato che divide la strada dal naviglio della Martesama.

Those desperate to cross the border would have paid around 150 euros each. The drivers of the vehicles having been found out by the police left the vehicles on the highway, with the illegal immigrants on board, on a slope and against the traffic. An open-air refugee camp has now been created at the end of via Sammartini, next to Milan Central Station, where there is the municipal hub to welcome migrants. There are at least a hundred refugees and asylum seekers who camp at night - but now also during the day - and sleep on the sidewalks and on the lawn that divides the road from the Martesama canal. (doc#1362; 7/9/2016, Repubblica).^15^

Finally, the extracts from the Italian news portals comprise nominations, such as *The refugee emergency, the continuous arrivals* (extract 6), *The bulletin of despair* (extract 7), *illegal immigrants on board*, *at least a hundred refugees and asylum seekers* (extract 8), which accelerate a sense of burden that the host societies need to undertake because of the construed emergency. Once more, an argumentation in favour of counteractions for the prevention of the migratory phenomenon is underpinned. This can be outlined in terms of the DHA *topos of burdening* or *weighing down*, which “is to be regarded as a specific causal topos (a topos of consequence)” and could “be reduced to the following conditional: if […] a ‘country’ is burdened by specific problems, one should act in order to diminish these burdens” (see Reisigl and Wodak 2001, 78).^16^

## Discussion: Conclusions

To summarize our findings, following the rationale of studies belonging to CL, the first layer of our micro-level analysis (i.e., frequency and keywords analyses) has shown that the news portals under study construed the mobilization of refugee migrant populations as a focal issue both for national and (extra)European affairs; this transpires from the presence of nominal types that refer to national contexts related to migration (i.e., *country, island, Italy, Libya, Turkey*) along with others that pinpoint the European dimension of the issue (e.g., *European, EU*). In doing so, the construction of a highly polarized national/ European discourse is favoured by the presence of lemmas that promote a humanitarian perspective (e.g., *hospitality, asylum*) vis-à-vis others that underpin a more discriminatory and preventive one (e.g., *detention, border, police*) in open/close spatial representations of Europe.

Focusing, then, on concordances of migrant* and refugee* in our corpora, in most cases, we were able to show how migrant populations are discursively mobilized in our corpora. Specifically, we witnessed instantiations in which the media framed relevant populations and the activities in which they are involved within a clear sense of threat and urgency. Crucially, this was logically linked to potential consequences for the host societies from the alleged massive number of migrants, with frequent depictions of ‘burden’. We thus, identified three main patterns that we qualitatively analysed through the analytical prism of the DHA *nomination-, predication-* and *argumentation strategies*. In doing so, we have shown that possible standpoints for the prevention of migration emerge on the basis of three main topoi, namely topoi of *danger/threat*, *numbers* and *burdening* or *weighing down*. Moreover, since this argumentative dynamic (i.e., preventing migration on the basis of the identified topoi) seems to continuously permeate online news portals’ discourses both from the centre-right and the centre-left across different social settings (i.e., Greece, Malta and Italy), we can reasonably argue that a macro-level anti-migrant discriminatory/xenophobic attitude is argumentatively justified and therefore established in the Mediterranean mediascape. According to the respective lines of reasoning, migrant populations are construed as ‘Others’ that should be discriminated and/or hated by host populations because they constitute a menacing burden to host societies. These reasoning lines constitute the logical basis that can potentially justify EU/European policies of restriction and exclusion based on the denigrating, discriminatory attitudes they cultivate against migrant populations; this is the argumentative basis of (soft) hate speech in the examined timeframe and contexts.

Specifically, although none of the examined discursive instantiations falls under the definitions of prosecutable hate speech, since they do not explicitly incite to violence or hatred against them (see also Sect. 1), they reify discursive-argumentative constructions, that “raise[…] serious concerns in terms of intolerance and discrimination” (Baider et al. 2017, 4) as they tend to consolidate and escalate in the mediascape (e.g., van Dijk 1991; Richardson 2004; Baker et al. 2013). From this perspective, following Baider et al. (2017, 3), the lines of reasoning (i.e., *topoi*) identified in Sect. 5, “may have a devastating effect on their recipients” since they can trigger and/or legitimise (unexpressed) hatred claims against them (see also Serafis 2022).These views, in turn, can constitute the basis on which different anti-migrant and/or hatred standpoints can be further legitimised and institutionalized as ‘necessary actions’.

As discussed in Sect. 2, since mass media are among the main actors that set the agenda in a *networked public sphere*, by privileging specific constructions of social reality, and interrelating with policy makers and other stakeholders, they can contribute to legitimise political decision-making processes and agendas. Throughout a two-year period at the centre of the so-called ‘refugee crisis’, the anti-migrant constructions favoured by the news portals under study and the argumentative dynamic that these constructions underpin can frame and justify a xenophobic ‘reality’ within which the migrant- ‘Other’ can legitimately be the potential target of discriminatory and hatred comments and actions. In its turn, this framing can justify the development of new policy-making that will be honed in on several discriminatory and hatred claims on the basis of the topoi of *danger/threat*, *numbers* and *burdening/weighing down*. In this sense, anti-migrant politics of hatred can be institutionalised through mainstream media discourses, deepening social inequalities in ‘refugee crisis’-hit Mediterranean.

What we hope to have shown in this paper is that the systematic analysis of mass media discourses through the methodological lens outlined in this paper can be a first necessary step towards the establishment of a rigorous and critical scholarly stance against social inequalities that certain discursive patterns may aggravate in highly polarised contexts. It is worth mentioning that our effort does not exist in limbo. In their interdisciplinary tradition, CDS have facilitated ways of scholarly critique. For example, Reisigl and Wodak (2001, 32–35) have distinguished between three main types of such an endeavour, namely (a) *text or discourse immanent critique*, (b) *sociodiagnostic critique* and (c) *prospective/retrospective critique*.

According to them, *text or discourse immanent critique* “[…] is based on a hermeneutic exegesis with the help of specific linguistic and discourse-analytical tools [;] [it] aims at discovering inconsistencies, (self-)contradictions, paradoxes and dilemmas in the text-internal or discourse internal […] structures” (Reisigl and Wodak 2001, 32). Our analysis, following a CL and DHA perspective, contributed to this direction by mapping the main meaningful attitudes in our corpora and discursive-argumentation strategies on the basis of which denigrating attitudes against migrants can be established by specific textual choices that online news portals employ to construe migration, all the while legitimising an anti-immigrant, discriminatory stance against migrant populations. Future research could follow these lines of research to delve into an analysis of argumentative inferences that stem from specific topoi (see Serafis et al. 2021a, b; Serafis and Assimakopoulos in press) or, perhaps even identify the weak and/or fallacious nature of such claims (Boukala 2021). The pragma-dialectical *rules for critical discussion* (e.g., van Eemeren and Grootendorst 2003, 2004) could fulfil this endeavour (see also Forchtner and Tominc 2012). In this effort, studies that emphasise the emotions (pathos) that are triggered by certain discursive wrappings (see Herman and Serafis 2019; Zappettini et al. 2021) can strengthen this angle of critique on covert forms of hate speech.

Moreover, this first angle of critique can further *sociodiagnostic critique*. This “aims at detecting problematic […] social and political goals and functions of discursive practices […] which are either inferable from the (spoken or written) discourse itself or from contextual, social, historical and political knowledge” (Reisigl and Wodak 2001, 32–33). Though that lens, “the analyst exceeds the purely textual or discourse internal sphere [and] makes use of her or his background and contextual knowledge and embeds the communicative or interactional structures of a discursive event in a wider frame of social and political relations, processes and circumstances” (Reisigl and Wodak 2001, 32–33). Our analysis enables us to put together theoretical insights from *networked publics* from an *institutional perspective* and pointed out *self-reinforcing and path-dependent discursive chains* that as a whole can plausibly illustrate a way in which media can discursively motivate discriminatory/hateful policy-making and establish new exclusionary ‘realities’ in societies (see Krzyżanowski 2020a, b; Krzyżanowski and Krzyżanowska 2022). To further establish a sociodiagnostic critique, the notion of *validity claims* that Habermas’s (1984, 99) develops in *The Theory of Communicative Action* could motivate future studies along the lines described here (see Forchtner 2011 for a detailed synthesis through the prism of CDS).

Finally, *prospective/retrospective critique* “seeks to become practical and to change and transform things” (Reisigl and Wodak 2001, 33ff) by actively participating in policy-making networks and consultancies, raising awareness events with employees in NGOs, school teachers and professors as well as journalists that can facilitate networks of practices against discrimination and disseminate resistance against social inequalities that are reproduced by xenophobic rhetoric and established through hatred politics. This last goal could not be fulfilled by a single study, such as the present one, but it presupposes a synthesis of a plethora of actively and socially motivated ways of doing scholarship that goes beyond strict academic boundaries and engages with social practices and action as a whole. Yet, a detailed scrutiny of dominant discourses in the public sphere is undoubtedly a necessary first step in this direction.

## Footnotes

1 While in some cases articles revolved around reported speech, we still assume the editorial intention to framing events from specific evaluative angles; thus, by reproducing a specific institutional discourse, the media can effectively be taken to give further legitimate value to those very discursive logics. We also recognise an industry self-regulatory code of practice exists whereby journalists should adhere to appropriate terminology (e.g. ‘immigrant’ vs. ‘asylum seeker’).

2 Newsbrief platform is publicly available at: https://emm.newsbrief.eu/NewsBrief/clusteredition/el/latest_en.html (last accessed: 18/02/2022).

3 See: https://auth.sketchengine.eu/#login (last accessed: 18/02/2022).

4 Translations into English were made by the authors.

5 In fact, Malta and Italy have been long dealing with migrant populations coming from Africa (see Schumacher 2020) which were further amplified by the ‘refugee crisis.’ See, for example, the case of a shipwreck in 2013: https://www.saccuccipartners.com/en/2021/01/28/italia-condannata-per-il-naufragio-del-2013-in-sar-maltese/ (last accessed: 28/07/2022).

6 Probably a consequence of the fact that in Italy illegal immigration is considered a crime. See more at: https://www.altalex.com/documents/leggi/2012/04/18/pacchetto-sicurezza-introdotto-il-reato-di-immigrazione-clandestina (last accessed: 10/08/2022).

7 As evident by recent critical discourse-analytical studies, this is typical of migration discourse (see e.g., Musolff 2015).

8 See: https://www.protothema.gr/politics/article/481256/-patoulis-ethniko-shedio-diaheirisis-tou-metanasteutikou-provlimatos/ (last accessed: 24/03/2022).

9 See: https://www.efsyn.gr/ellada/dikaiomata/28626_ta-lathro-reportaz-kai-i-alithini-eikona (last accessed: 24/03/2022).

10 See: https://www.efsyn.gr/ellada/dikaiomata/33390_nea-kinitopoiisi-prosfygon-sti-lesbo (last accessed: 24/03/2022).

11 See: https://www.independent.com.mt/articles/2015-06-19/world-news/European-Union-to-launch-operation-against-Libya-migrant-smugglers-6736137573#bmComment (last accessed: 24/03/2022).

12 See: https://www.maltatoday.com.mt/news/europe/56133/mediterranean_migrant_arrivals_approach_250000__iom#.YjyRe-dBw2w (last accessed: 24/03/2022).

13 See: https://bergamo.corriere.it/notizie/cronaca/15_luglio_29/cento-profughi-via-palestre-pronte-riaprirsi-porte-gleno-4fe63c44-35cd-11e5-b050-7dc71ce7db4c.shtml (last accessed: 24/03/2022).

14 See: https://genova.repubblica.it/cronaca/2015/07/03/news/viale_lega_doria_firmi_la-118243244/?rss (last accessed: 24/03/2022).

15 See: https://milano.repubblica.it/cronaca/2016/07/09/news/profughi_milano-143753225/?rss (last accessed: 24/03/2022).

16 In some cases, a *topos of numbers* is also implied.
